# The association between weight-adjusted waist index and sleep disorders in U.S. adults: results from NHANES 2005–2008

**DOI:** 10.1097/MD.0000000000039589

**Published:** 2024-09-13

**Authors:** Jiayun Zheng, Yue Xi, Hang Jiang

**Affiliations:** a The First Clinical Medical School, Guangzhou Medical University, Guangzhou, China.

**Keywords:** cross-sectional study, NHANES (National Health and Nutrition Examination Survey), sleep disorders, United States, weight-adjusted waist index

## Abstract

The detrimental effects of obesity on sleep disorders have garnered a lot of interest. The weight-adjusted waist index (WWI) is a newly developed anthropometric index calculated in terms of weight and waist circumference. The body mass index has been employed to evaluate obesity in the majority of studies that connect obesity to sleep disorders. This study seeks to investigate the correlation between WWI and sleep disorders among adults in the United States. This cross-sectional study was part of the National Health and Nutrition Examination Survey and included adults aged >20 from 2005 to 2008. This study investigated the linear relationship between sleep disorders and WWI using weighted binary logistic regression models. Nonlinear relationships were characterized using smooth curve fitting and threshold effects analyses. After that, based on variables like gender, age, marital status, diabetes, hypertension, and smoking, subgroup analyses were performed. Our study included 9869 participants who were at least 20 years old. Higher WWI was linked to greater odds of sleep disorders prevalence, according to weighted binary logistic regression (odds ratio = 1.15; 95% confidence interval, 1.10, 1.20). In subgroup analyses based on age, marital status, diabetes, hypertension, and smoking, this connection remained robust. However, there were notable differences in this connection depending on gender. Furthermore, a nonlinear correlation with inflection points between WWI and sleep disorders was shown using smooth curve fitting. The nonlinear association between WWI and sleep disorders has an inflection point of 8.1 cm/√kg, as indicated by the threshold effect analyses. A higher WWI exposure may elevate the odds of sleep disorder prevalence, underscoring the importance of considering WWI in the prevention and management of sleep disorders.

## 1. Introduction

Sleep is essential for all human activities. About one-third of a person’s life is spent sleeping. Numerous behavioral and physiological systems depend on sleep, and when these processes are disrupted, it can result in a variety of sleep disorders and eventually physiological dysfunction.^[1]^ The Centers for Disease Control classifies sleep disorders as a public health issue.^[2]^ In the general population, sleep disorders are strongly linked to higher death rates, metabolic diseases, atherosclerosis, cardiovascular disease, and lower standards of life.^[3,4]^

Obesity is becoming more commonplace worldwide, and being overweight or obese has become a major public health concern.^[5]^ A body mass index (BMI) of more than 25 kg/m^2^ is classified as overweight and more than 30 kg/m^2^ is classified as obese.^[6]^ Numerous studies have revealed that it heightens the risk of various common diseases, including cancer, diabetes, heart disease, and infertility in women.^[7–9]^ Furthermore, obesity negatively affects sleep due to the development of obstructive sleep apnea, obesity hypoventilation syndrome, and the release of inflammatory cytokines that interfere with sleep cycles.^[10,11]^

BMI has been used to evaluate obesity in previous research. However, because it ignores variations in bone density, fat distribution, and muscle mass, its validity as a crude indicator of obesity has been called into question.^[12]^ Waist circumference (WC), on the other hand, has a substantial link with the accumulation of abdominal fat, making it a stronger indication of the health effects of obesity.^[13]^ The weight-adjusted waist index (WWI), which is computed by dividing WC (cm) by the square root of body weight (kg) (cm/√kg), is a novel obesity index that researchers have just presented.^[14]^ The index represents weight-independent central obesity while accounting for body weight.^[15]^ It is a more reliable indicator of cardiovascular disease morbidity and mortality than BMI, body shape index, and waist-to-height ratio, according to earlier research.^[14,16]^ Additionally, WC and BMI were not as good predictors of hypertension as WWI was.^[17]^ According to several research, WWI is a more accurate measure than BMI.^[18–20]^

Research has indicated that obesity is linked to many sleep disorders, such as obstructive sleep apnea, insomnia, and restless legs.^[21–23]^ There is little research on the connection between WWI and sleep disorders, even though WWI can be used as a marker of central obesity. Consequently, to deepen our understanding of this field, we evaluated the relationship between WWI and sleep disorders in individuals whose sleep disorders were investigated.

## 2. Methods

### 2.1. Study population

**Figure 1.**
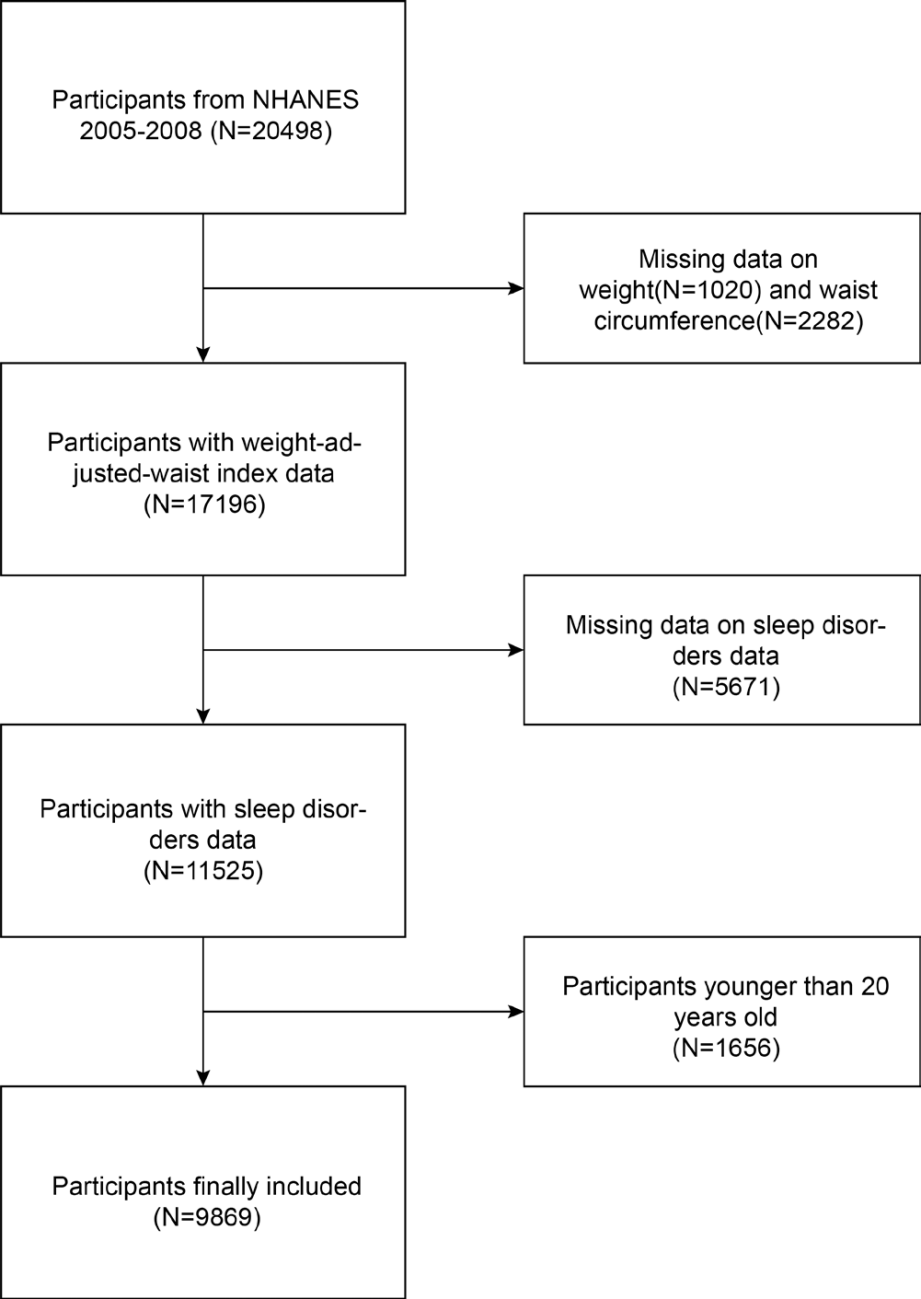
Flowchart showing the NHANES 2005–2008 sample selection process. NHANES = National Health and Nutrition Examination Survey.

The National Center for Health Statistics conducts National Health and Nutrition Examination Survey (NHANES) research to investigate the health and nutritional status of the US population. All NHANES data is publicly accessible at https://www.cdc.gov/nchs/nhanes/. The NHANES sample is representative due to the stratified multistage probability sampling method employed in the research design. Data for this cross-sectional study was contributed by NHANES participants aged over twenty who participated in the 2005–2008 cycle. Throughout the 2005–2006 and 2007–2008 periods, 20,498 people took part in NHANES, with no data on body weight (n = 1020), WC (n = 2282), sleep disorders (n = 5671), and those under the age of 20 (n = 1656) excluded (Fig. 1).

### 2.2. Exposure and ending definition

WWI (cm/√kg) was the exposure variable in this study. The presence or absence of a sleep disorder was the targeted independent variable. Sleep disorders were covered by NHANES questions SLQ050 and SLQ060, which asked: “Ever told by the doctor have a sleep disorder?,” “Ever told doctor had trouble sleeping?” The response of “yes” indicated the presence of a sleep disorder. SLQ070: Self-reporting of insomnia, restless legs, sleep apnea, or other sleep disorders. Following their response, those who replied “yes” were then deemed to have a sleep disorder.^[24]^

### 2.3. Covariates

Based on reports in the literature,^[25–30]^ covariates that could potentially influence the link between WWI and sleep disorders were integrated into our survey design. The age and ratio of poverty-to-income ratio were continuous variables. Gender, race, education level, marital status, hypertension, diabetes, smoking, and alcohol consumption were categorical variables. Individuals are classified as smokers if they smoke more than 100 cigarettes in their lifetime, and drinkers if they drink more than 12 times a year in their lifetime. Hypertension and diabetes are defined based on participants’ responses to the questions, “Has a doctor ever told you that you have hypertension?” and “Has a doctor ever told you that you have diabetes?” respectively. These factors were all taken from NHANES questionnaire data and demographic data. The public can see all their detailed measurement protocols at https://www.cdc.gov/nchs/nhanes/.

### 2.4. Statistical analyses

Following the guidelines outlined by the Centers for Disease Control and Prevention, we conducted all statistical analyses utilizing R (http://www.r-project.org) and EmpowerStats (http://www.empowerstats.com). The threshold for statistical significance is set at *P* < .05. This study applied the National Center for Health Statistics analytic guidelines to generate estimates and used sample weights aligned with NHANES objectives, ensuring the study’s representativeness. Categorical variables were expressed as percentages, while continuous data were presented as means and standard deviations. To evaluate differences across WWI triads, weighted Student’s *t* tests were used for normally distributed continuous variables, Kruskal-Wallis test for non-normally distributed continuous variables, and weighted chi-square tests for categorical variables. The study utilized weighted multiple logistic regression to investigate the independent association between WWI and sleep disorders across 3 models. To explore the nonlinear relationship, smooth curve fitting and threshold effects analyses were conducted, complemented by subgroup analyses that considered gender, age, marital status, diabetes, hypertension, and smoking.

## 3. Results

### 3.1. Baseline characteristics of the study population

There were 9869 individuals in this study aged at least 20 years. The participants’ average age was 49.04 ± 18.16 years, with 51.56% of them were female and 48.44% of them were male. The majority of the population were White individuals, with 48.12%, followed by Non-Hispanic Black individuals (21.52%), Mexican Americans (18.72%), Other Hispanic individuals (7.57%), and other races (4.07%). The average WWI was 8.09 ± 1.47 cm/√kg. 24.11% experienced problems with their sleep. Table 1 lists the individuals’ clinical characteristics, and all individuals were evenly separated into 3 groups based on WWI using column-stratified grouping. The tertiles are demarcated as follows: Tertile 1 ranges from 4.11 to 7.37, Tertile 2 from 7.37 to 8.58, and Tertile 3 from 8.58 to 17.65. Compared to Tertile 1, the highest WWI tertile (Tertile 3) was associated with greater rates of diabetes and hypertension, as well as being male, non-Hispanic White, and having a higher family income level.

**Table 1:** The research population’s baseline characteristics based on the weight-adjusted waist index tertile.

Weight-adjusted waist index	T1 (4.11–7.37)	T2 (7.37–8.58)	T3 (8.58–17.65)	*P* value
	N = 3290	N = 3288	N = 3291	
Age (yr)	50 (35)	48 (30)	46 (25)	<.001
Gender (%)				
Male	825 (25.08)	1770 (53.83)	2186 (66.42)	<.001
Female	2465 (74.92)	1518 (46.17)	1105 (33.58)	
Race (%)				
Mexican American	780 (23.71)	662 (20.13)	405 (12.31)	<.001
Other Hispanic	320 (9.73)	240 (7.30)	187 (5.68)	
Non-Hispanic White	1509 (45.87)	1600 (48.66)	1640 (49.83)	
Non-Hispanic Black	464 (14.10)	687 (20.89)	973 (29.57)	
Other	217 (6.60)	99 (3.01)	86 (2.61)	
Education level (%)				
Less than 9th Grade	581 (17.66)	449 (13.66)	222 (6.75)	<.001
9–11th Grade	576 (17.51)	528 (16.06)	518 (15.74)	
High School Grad/GED or Equivalent	796 (24.19)	774 (23.54)	830 (25.22)	
Some College or AA degree	747 (22.71)	883 (26.86)	1049 (31.87)	
College Graduate or above	590 (17.93)	654 (19.89)	672 (20.42)	
Marital status (%)				
Married	1900 (57.75)	2100 (63.87)	2115 (64.27)	<.001
Divorced	889 (27.02)	676 (20.56)	606 (18.41)	
Never married	501 (15.23)	512 (15.57)	570 (17.32)	
Ratio of family income to poverty	2.14 (2.40)	2.24 (2.66)	2.49 (3.03)	<.001
Hypertension (%)				
Yes	932 (28.33)	1047 (31.84)	1317 (40.02)	<.001
No	2358 (71.67)	2241 (68.16)	1974 (59.98)	
Diabetes (%)				
Yes	295 (8.97)	334 (10.16)	464 (14.10)	<.001
No	2995 (91.03)	2954 (89.84)	2827 (85.90)	
Smoking (%)				
Yes	1430 (43.47)	1618 (49.21)	1622 (49.29)	<.001
No	1860 (56.53)	1670 (50.79)	1669 (50.71)	
Alcohol intake ≥ 12 drinks/yr (%)				
Yes	2213 (67.26)	2396 (72.87)	2478 (75.30)	<.001
No	1077 (32.74)	892 (27.13)	813 (24.70)	
Sleep disorders (%)				
Yes	752 (22.86)	712 (21.65)	915 (27.80)	<.001
No	2538 (77.14)	2576 (78.35)	2376 (72.20)	

Mean + SD for normally distributed continuous variables. Med (IQR) for non-normally distributed continuous variables; *P* value for normally distributed continuous variables was calculated by subsequent students’ *t* test; *P* value for non-normally distributed continuous variables was calculated by the Kruskal-Wallis test.

% for Categorical variables: *P* value was calculated by the weighted chi-square test.

### 3.2. The occurrence of sleep disorders is correlated with WWI

Table 2 indicates that WWI and sleep disorders are positively correlated. The association remained unchanged in the fully adjusted model (Model 3) (odds ratio [OR] = 1.15; 95% confidence interval [CI] 1.10, 1.20). According to the study, the odds of suffering from sleep disorders increased by 15% for every unit increase in WWI. We also transformed WWI into a tri-categorical variable from a continuous one. Tertile 3 had odds of sleep disorder prevalence that were 37% greater than those of Tertile 1, which was found in the fully adjusted model (OR = 1.37; 95% CI 1.12, 1.68). However, Tertile 2 did not have any statistically significant variation compared to Tertile 1 (OR = 1.03; 95% CI 0.83, 1.28).

**Table 2:** Association between WWI and sleep disorder.

	OR (95% CI), *P* value		
	Model 1	Model 2	Model 3
WWI (continuous)	1.09 (1.05, 1.13), <.01	1.19 (1.14, 1.24), <.01	1.15 (1.10, 1.20), <.01
WWI (tertile)			
Tertile 1	Reference	Reference	Reference
Tertile 2	0.90 (0.76, 1.06), .21	1.08 (0.89, 1.31), .43	1.03 (0.83, 1.28), .78
Tertile 3	1.15 (0.99, 1.34), .09	1.56 (1.29, 1.88), <.01	1.37 (1.12, 1.68), <.01

The WWI was transformed from a continuous to a categorical variable (tertiles) for sensitivity analysis.

Model 1: No covariates were adjusted. Model 2: Adjusted for gender, age, and race. Model 3: Adjusted for gender, age, race, education level, ratio of family income to poverty, marital status, hypertension, diabetes, smoking, and alcohol intake ≥12 drinks/yr.

95% CI = 95% confidence interval, OR = odds ratio, WWI = weight-adjusted waist index.

### 3.3. Smooth curve fitting

**Figure 2.**
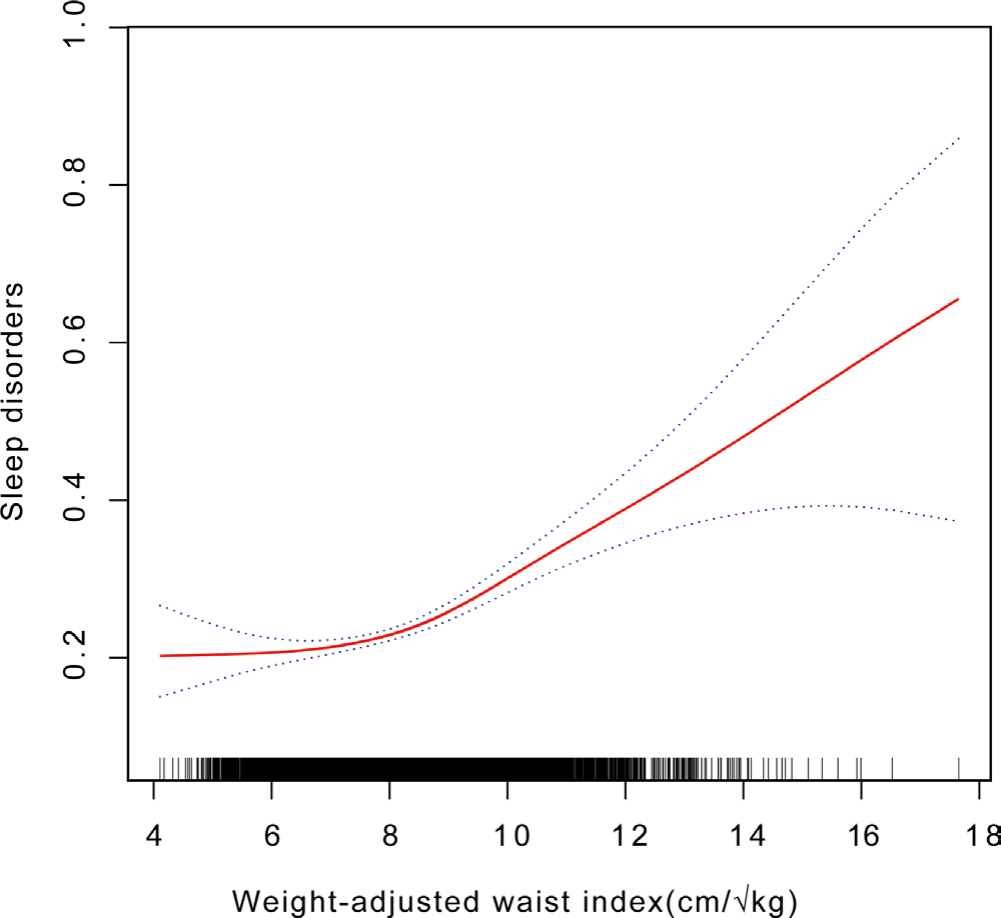
The smooth curve fitting analyses of WWI and sleep disorders. The solid red line is the representation of the smooth curve between the variables. The blue band represents the results, which are 95% confidence intervals adjusted for gender, age, race, marital status, education level, household income to poverty ratio, smoking, alcohol use, diabetes, and hypertension. WWI = weight-adjusted waist index.

The non-linear relationship between WWI and sleep disorders was studied through the use of smooth curve fitting. In Figure 2, the positive non-linear relationship between WWI and sleep disorders is demonstrated.

### 3.4. Analyses of the threshold effect of WWI on the odds of sleep disorders prevalence

We found that there was an inflection point of 8.1 cm/√kg for sleep disorders by an investigation of the threshold influence of WWI on sleep disorders (Table 3). To the right of the inflection point, there was a positive correlation between WWI and sleep disorders (OR = 1.24; 95% CI 1.18, 1.31), while there was no statistically significant correlation to the left of the inflection point (OR = 1.03; 95% CI 0.96, 1.11). To put it another way, there was not a significant connection between WWI and sleep disorders until WWI reached 8.1 cm/kg. Sleep disorders were found to be strongly correlated with an increase in WWI once it reached 8.1 cm/kg. For every unit rise in WWI, there was a 24% increase in the odds of sleep disorders prevalence.

**Table 3:** Analyses of the threshold effect of WWI on the odds of sleep disorder prevalence.

Sleep disorders	Adjusted OR (95% CI), *P* value
Fitting by the standard linear model	1.16 (1.12, 1.20), <.01
Fitting by the 2-piecewise linear model	
Inflection point	8.1
Weight-adjusted waist index <	1.03 (0.96, 1.11), .4215
Weight-adjusted waist index >	1.24 (1.18, 1.31), <.01
*P* for Log-likelihood ratio	<0.01

Adjusting for gender, age, race, education level, PIR, marital status, hypertension, diabetes, smoking, and alcohol intake ≥ 12 drinks/yr, the results of the threshold effect of WWI on the odds of sleep disorders prevalence were obtained.

PIR = poverty-to-income ratio, WWI = weight-adjusted waist index.

### 3.5. Subgroup analyses

Stratified by gender, age, marital status, diabetes, hypertension, and smoking, subgroup analyses, and interaction tests were carried out to assess the robustness of the association between WWI and sleep disorders and to pinpoint possible population variances (Table 4). The subgroup analyses revealed that there was a significant correlation (all *P* < .05) between WWI and greater odds of sleep disorder prevalence in each of the subgroups stratified. Significant gender-based variations were found in the interaction tests examining the relationship between WWI and sleep disorders. As WWI increased, the odds of sleep disorder prevalence were higher in the male group than in the female group. There was no significant difference between the rest of the strata, which suggests that age, marital status, diabetes, hypertension, and smoking were stable in the relationship between WWI and sleep disorders.

**Table 4:** Subgroup analyses.

Sleep disorders	OR (95% CI)	*P* for interaction
Gender (%)		
Male	1.24 (1.17, 1.30)	.0021
Female	1.11 (1.06, 1.16)	
Age (yr)		
<60	1.11 (1.07, 1.16)	.3170
≥60	1.16 (1.09, 1.23)	
Marital status (%)		
Married with partner	1.19 (1.14, 1.25)	.0636
Divorced	1.10 (1.02, 1.17)	
Never married	1.11 (1.02, 1.20)	
Diabetes (%)		
Yes	1.16 (1.06, 1.26)	.9993
No	1.16 (1.11, 1.20)	
Hypertension (%)		
Yes	1.13 (1.07, 1.19)	.4472
No	1.16 (1.10, 1.21)	
Smoking (%)		
Yes	1.14 (1.09, 1.20)	.4570
No	1.17 (1.12, 1.23)	

95% CI = 95% confidence interval, OR = odds ratio.

## 4. Discussion

This study aimed to investigate the relationship between WWI and sleep disorders among US adults. Our cross-sectional survey of 9869 adults revealed a correlation between greater levels of WWI and sleep disorders. Investigated from the inflection point’s left and right sides (WWI = 8.1 cm/√kg), the right side showed a significant relationship with the odds of sleep disorders prevalence, but the left side did not show a statistically significant relationship. The findings of this study imply that lowering WWI levels may help lower the prevalence of sleep disorders.

Research has now demonstrated that obesity is a significant risk factor and a predictor of unfavorable clinical outcomes.^[31]^ Diabetes, hypertension, dyslipidemia, and sleep disorders are all linked to obesity.^[11,32–34]^ BMI is currently the accepted measure for screening for obesity. However, because BMI measures extra weight rather than body fat, it only partially represents obesity. BMI does not take into account the adipose tissue distribution of major central fat deposits around the neck, trunk, and abdominal viscera, but the regional distribution of body fat as measured by waist and neck circumference is a better determinant of the severity of OSA than is generalized obesity.^[35]^ BMI is an insufficient biomarker of abdominal obesity, however, WC is a straightforward approach to measuring abdominal obesity that can be standardized and applied clinically.^[15]^ Therefore, our team decided to use the WWI index—which is derived from weight and WC—as the screening indication given the significance of the completeness of the evaluation of obesity.

This study is the first to focus on the association between WWI and sleep disorders. Previous research has shown that obesity has a negative impact on sleep quality, including obesity hypoventilation syndrome and OSA.^[10]^ Obesity raises the risk of OSAS, which is probably caused by excess adipose tissue constricting the upper airway.^[36]^ Obesity also causes abnormal ventilation-perfusion ratios, reduced lung capacity, and restricted lung and chest wall motion.^[36]^ Sleep apnea resulting from OSAS can produce intermittent hypoxia and hypercapnia, which can cause sleep disorders such as fragmented sleep, recurrent nocturnal arousals, and enhanced respiratory effort.^[37]^ Obesity enlarges the amount and size of pharyngeal fat deposits, which is a major contributor to respiratory compression. The development of OSAS may be influenced by fat deposits in the upper airway and the area around the thoracic cavity.^[38]^ Furthermore, elevated visceral adipose tissue may secrete inflammatory cytokines including TNF-α, IL-6, and IL-1, which might interfere with sleep-wake cycles.^[39,40]^ The 2 most researched cytokines linked to obesity are TNF-a and IL-6, which are found in higher concentrations in the adipose tissue and serum of obese individuals.^[41]^ Research has demonstrated heightened somnolence in obese participants without sleep disorders. This could be attributed to the impact of IL-6 generated by obesity-related adiposity.^[42]^ A clinical study’s findings indicated that obese individuals who had shed pounds had decreased levels of TNF-a and IL-6 as well as improved sleep duration and quality.^[43]^

In one study, weight loss and lifestyle modifications led to sustained improvements in the severity of OSA and associated comorbidities. Spanish men who were overweight or obese and received CPAP treatment for moderate to severe OSA demonstrated improvement in both their hepatic and cardiovascular functioning, respectively.^[44]^ In a different study, throughout the very low-calorie ketogenic diet-induced weight reduction, improvements in sleep indices were noted, particularly during the maximum loss of adiposity.^[45]^ According to a study by Shade et al, women who lost 5 percent or more of their body weight experienced improvements in blood pressure, pain interference, and sleep quality. Weight reduction also lessens discomfort and sleep disturbance, and women who experience fewer sleep disruptions may also have lower blood pressure.^[46]^ Furthermore, the research has demonstrated a significant correlation between the amount of sleep and WC, suggesting that sleep deprivation and increased central adiposity can co-occur.^[47]^ A 1-centimeter increase in WC represents a percentage of increased risk for mild, moderate, and severe OSA.^[48]^

However, as academic research progressed using BMI and WC as indicators of obesity, the obesity paradox emerged. According to research by Rahman et al,^[49]^ calcification of the abdominal aorta was negatively correlated with an increase in BMI. According to Park et al,^[14]^ survivors all had mean BMI that were unexpectedly higher than those of decedents who died from cardiovascular or all-cause mortality. The obesity paradox has brought into question the validity of BMI as an indicator of obesity in academia. WWI is a relatively new measurement tool, and recent studies have shown that it can distinguish between muscle mass and adiposity. As a result, its application has expanded to include a range of conditions, such as female reproduction, psychiatric disorders, renal disease, cardiovascular disease, and mental health issues.^[18,49–54]^

The main advantages of this study are that it is the initial cross-sectional analyses to examine the connection between WWI and sleep disorders, and it has a sizable and representative sample size. The findings are broadly applicable to the entire US population because we used national data and took sample weights into account in our study. Covariate adjustments were made to the regression models, and robustness was confirmed by subgroup analyses made possible by the high sample size. Furthermore, WWI is capable of analyzing both muscle and fat mass and is not restricted to analyzing fat content in specific regions. Instead, it considers visceral fat and WC, yielding more complete findings. However, there are several shortcomings in our study. Firstly, in order to ascertain if a causal association exists between WWI and sleep disorders, more study is necessary as the cross-sectional survey was unable to determine it. Secondly, there was a dearth of externally validated sleep disorders in the study, such as the PSQI scale, the ESS scale, and the DBAS scale. A memory bias was introduced because the diagnosis of sleep disorders was solely based on participant self-reports. Thirdly, several possible confounders may have had an impact on WWI and sleep disorders. Even with the inclusion of quite a few pertinent factors in our model, it is still difficult to eliminate the impact of additional possible confounding variables. Fourthly, as our research is limited to a single nation and ethnic group, it is necessary to determine whether the results apply to other nations or ethnic groups.

## 5. Conclusion

According to our research, a higher level of WWI was linked to larger odds of sleep disorder prevalence. Thus, high WWI may be a potential risk for sleep disorders. To avoid jeopardizing their health, those with greater WWI should pay more attention to their sleep health. However, further large-scale prospective investigations are required to bolster the findings presented here.

## Acknowledgments

The authors would like to thank the NHANES database for providing useful datasets for upload.

## Author contributions

**Conceptualization:** Jiayun Zheng, Yue Xi.

**Data curation:** Jiayun Zheng, Yue Xi.

**Formal analysis:** Jiayun Zheng, Hang Jiang.

**Investigation:** Jiayun Zheng, Yue Xi.

**Methodology:** Jiayun Zheng, Hang Jiang.

**Software:** Jiayun Zheng.

**Supervision:** Jiayun Zheng, Yue Xi.

**Validation:** Jiayun Zheng.

**Writing – original draft:** Jiayun Zheng, Yue Xi, Hang Jiang.

**Writing – review & editing:** Jiayun Zheng.

## References

[1] Sateia MJ. International classification of sleep disorders-third edition. Chest. 2014;146:1387–94. 25367475 10.1378/chest.14-0970

[2] Watson NF Badr MS Belenky G.; Consensus Conference Panel. Joint consensus statement of the American Academy of sleep medicine and sleep research society on the recommended amount of sleep for a healthy adult: methodology and discussion. Sleep. 2015;38:1161–83. 26194576 10.5665/sleep.4886 PMC4507722

[3] Summa KC Turek FW. Chronobiology and obesity: interactions between circadian rhythms and energy regulation. Adv Nutr. 2014;5:312S–9S. 24829483 10.3945/an.113.005132 PMC4013188

[4] Reutrakul S Van Cauter E. Sleep influences on obesity, insulin resistance, and risk of type 2 diabetes. Metabolism. 2018;84:56–66. 29510179 10.1016/j.metabol.2018.02.010

[5] Ng M Fleming T Robinson M. Global, regional, and national prevalence of overweight and obesity in children and adults during 1980–2013: a systematic analysis for the Global Burden of Disease Study 2013. Lancet. 2014;384:766–81. 24880830 10.1016/S0140-6736(14)60460-8 PMC4624264

[6] Lin X Li H. Obesity: epidemiology, pathophysiology, and therapeutics. Front Endocrinol (Lausanne). 2021;12:706978. 34552557 10.3389/fendo.2021.706978 PMC8450866

[7] Piché ME Tchernof A Després JP. Obesity phenotypes, diabetes, and cardiovascular diseases. Circ Res. 2020;126:1477–500. 32437302 10.1161/CIRCRESAHA.120.316101

[8] Broughton DE Moley KH. Obesity and female infertility: potential mediators of obesity’s impact. Fertil Steril. 2017;107:840–7. 28292619 10.1016/j.fertnstert.2017.01.017

[9] Iyengar NM Gucalp A Dannenberg AJ Hudis CA. Obesity and cancer mechanisms: tumor microenvironment and inflammation. J Clin Oncol. 2016;34:4270–6. 27903155 10.1200/JCO.2016.67.4283 PMC5562428

[10] Meurling IJ Shea DO Garvey JF. Obesity and sleep: a growing concern. Curr Opin Pulm Med. 2019;25:602–8. 31589189 10.1097/MCP.0000000000000627

[11] Muscogiuri G Barrea L Annunziata G. Obesity and sleep disturbance: the chicken or the egg? Crit Rev Food Sci Nutr. 2019;59:2158–65. 30335476 10.1080/10408398.2018.1506979

[12] Bosello O Donataccio MP Cuzzolaro M. Obesity or obesities? Controversies on the association between body mass index and premature mortality. Eat Weight Disord. 2016;21:165–74. 27043948 10.1007/s40519-016-0278-4

[13] Fedewa MV Nickerson BS Esco MR. Associations of body adiposity index, waist circumference, and body mass index in young adults. Clin Nutr. 2019;38:715–20. 29653863 10.1016/j.clnu.2018.03.014

[14] Park Y Kim NH Kwon TY Kim SG. A novel adiposity index as an integrated predictor of cardiometabolic disease morbidity and mortality. Sci Rep. 2018;8:16753. 30425288 10.1038/s41598-018-35073-4 PMC6233180

[15] Qin Z Du D Li Y. The association between weight-adjusted-waist index and abdominal aortic calcification in adults aged ≥ 40 years: results from NHANES 2013-2014. Sci Rep. 2022;12:20354. 36437292 10.1038/s41598-022-24756-8 PMC9701694

[16] Ding C Shi Y Li J. Association of weight-adjusted-waist index with all-cause and cardiovascular mortality in China: a prospective cohort study. Nutr Metab Cardiovasc Dis. 2022;32:1210–7. 35277327 10.1016/j.numecd.2022.01.033

[17] Li Q Qie R Qin P. Association of weight-adjusted-waist index with incident hypertension: the Rural Chinese Cohort Study. Nutr Metab Cardiovasc Dis. 2020;30:1732–41. 32624344 10.1016/j.numecd.2020.05.033

[18] Li X Wang L Zhou H Xu H. Association between weight-adjusted-waist index and chronic kidney disease: a cross-sectional study. BMC Nephrol. 2023;24:266. 37691097 10.1186/s12882-023-03316-w PMC10494374

[19] Cao S Hu X Shao Y. Relationship between weight-adjusted-waist index and erectile dysfunction in the United State: results from NHANES 2001-2004. Front Endocrinol (Lausanne). 2023;14:1128076. 37181040 10.3389/fendo.2023.1128076 PMC10167952

[20] Sun D He H Luo B Xie B. The association between weight-adjusted-waist index and stress urinary incontinence in female adults: a population-based study. Int Urol Nephrol. 2024;56:1851–8. 38289545 10.1007/s11255-023-03928-z

[21] Masa JF Pépin JL Borel JC Mokhlesi B Murphy PB Sánchez-Quiroga MA. Obesity hypoventilation syndrome. Eur Respir Rev. 2019;28:180097. 30872398 10.1183/16000617.0097-2018 PMC9491327

[22] Drager LF Togeiro SM Polotsky VY Lorenzi-Filho G. Obstructive sleep apnea: a cardiometabolic risk in obesity and the metabolic syndrome. J Am Coll Cardiol. 2013;62:569–76. 23770180 10.1016/j.jacc.2013.05.045 PMC4461232

[23] De Vito K Li Y Batool-Anwar S Ning Y Han J Gao X. Prospective study of obesity, hypertension, high cholesterol, and risk of restless legs syndrome. Mov Disord. 2014;29:1044–52. 24753235 10.1002/mds.25860 PMC4501395

[24] Pei H Li S Su X Lu Y Wang Z Wu S. Association between triglyceride glucose index and sleep disorders: results from the NHANES 2005–2008. BMC Psychiatry. 2023;23:1–9. 36899383 10.1186/s12888-022-04434-9 PMC10007799

[25] Zhou T Chen S Mao J Zhu P Yu X Lin R. Association between obstructive sleep apnea and visceral adiposity index and lipid accumulation product: NHANES 2015-2018. Lipids Health Dis. 2024;23:100. 38600516 10.1186/s12944-024-02081-5 PMC11005189

[26] Cai S Li S Zhou Y Song J Peng J. The association between sedentary behavior and obstructive sleep apnea: a cross-sectional study from the NHANES (2007-2008 to 2015-2020). BMC Oral Health. 2024;24:224. 38347492 10.1186/s12903-024-03960-0 PMC10863124

[27] Wang S Lai F Zhao L. Association between vitamin C in serum and trouble sleeping based on NHANES 2017-2018. Sci Rep. 2024;14:9727. 38678062 10.1038/s41598-024-56703-0 PMC11055852

[28] Wen Z Li X. Association between weight-adjusted-waist index and female infertility: a population-based study. Front Endocrinol (Lausanne). 2023;14:1175394. 37614708 10.3389/fendo.2023.1175394 PMC10442810

[29] Wen SH Tang X Tang T Ye ZR. Association between weight-adjusted-waist index and gallstones: an analysis of the National Health and Nutrition Examination Survey. BMC Gastroenterol. 2024;24:40. 38238700 10.1186/s12876-024-03127-9 PMC10797852

[30] Ke B Sun Y Dai X Gui Y Chen S. Relationship between weight-adjusted waist circumference index and prevalence of gallstones in U.S. adults: a study based on the NHANES 2017-2020. Front Endocrinol (Lausanne). 2023;14:1276465. 37964952 10.3389/fendo.2023.1276465 PMC10641849

[31] Lavie CJ Alpert MA Arena R Mehra MR Milani RV Ventura HO. Impact of obesity and the obesity paradox on prevalence and prognosis in heart failure. JACC Heart Fail. 2013;1:93–102. 24621833 10.1016/j.jchf.2013.01.006

[32] Martyn JAJ Kaneki M Yasuhara S. Obesity-induced insulin resistance and hyperglycemia: etiologic factors and molecular mechanisms. Anesthesiology. 2008;109:137–48. 18580184 10.1097/ALN.0b013e3181799d45 PMC3896971

[33] Seravalle G Grassi G. Obesity and hypertension. Pharmacol Res. 2017;122:1–7. 28532816 10.1016/j.phrs.2017.05.013

[34] Vekic J Zeljkovic A Stefanovic A Jelic-Ivanovic Z Spasojevic-Kalimanovska V. Obesity and dyslipidemia. Metabolism. 2019;92:71–81. 30447223 10.1016/j.metabol.2018.11.005

[35] Kim NH Park Y Kim NH Kim SG. Weight-adjusted waist index reflects fat and muscle mass in the opposite direction in older adults. Age Ageing. 2021;50:780–6. 33035293 10.1093/ageing/afaa208

[36] Yaggi HK Strohl KP. Adult obstructive sleep apnea/hypopnea syndrome: definitions, risk factors, and pathogenesis. Clin Chest Med. 2010;31:179–86. 20488280 10.1016/j.ccm.2010.02.011

[37] Lv R Liu X Zhang Y. Pathophysiological mechanisms and therapeutic approaches in obstructive sleep apnea syndrome. Signal Transduct Target Ther. 2023;8:218. 37230968 10.1038/s41392-023-01496-3 PMC10211313

[38] Dempsey JA Veasey SC Morgan BJ O’Donnell CP. Pathophysiology of sleep apnea. Physiol Rev. 2010;90:47–112. 20086074 10.1152/physrev.00043.2008 PMC3970937

[39] Gamaldo CE Shaikh AK McArthur JC. The sleep-immunity relationship. Neurol Clin. 2012;30:1313–43. 23099140 10.1016/j.ncl.2012.08.007

[40] Hotamisligil GS. Inflammation and metabolic disorders. Nature. 2006;444:860–7. 17167474 10.1038/nature05485

[41] Cottam DR Mattar SG Barinas-Mitchell E. The chronic inflammatory hypothesis for the morbidity associated with morbid obesity: implications and effects of weight loss. Obes Surg. 2004;14:589–600. 15186624 10.1381/096089204323093345

[42] Bixler EO Vgontzas AN Lin HM Calhoun SL Vela-Bueno A Kales A. Excessive daytime sleepiness in a general population sample: the role of sleep apnea, age, obesity, diabetes, and depression. J Clin Endocrinol Metab. 2005;90:4510–5. 15941867 10.1210/jc.2005-0035

[43] Al-Sharif FM El-Kader SMA. Inflammatory cytokines and sleep parameters response to life style intervention in subjects with obese chronic insomnia syndrome. Afr Health Sci. 2021;21:1223–9. 35222585 10.4314/ahs.v21i3.31 PMC8843290

[44] Carneiro-Barrera A Amaro-Gahete FJ Guillén-Riquelme A. Effect of an interdisciplinary weight loss and lifestyle intervention on obstructive sleep apnea severity: the INTERAPNEA randomized clinical trial. JAMA Netw Open. 2022;5:e228212. 35452108 10.1001/jamanetworkopen.2022.8212 PMC9034401

[45] Castro AI Gomez-Arbelaez D Crujeiras AB. Effect of A very low-calorie ketogenic diet on food and alcohol cravings, physical and sexual activity, sleep disturbances, and quality of life in obese patients. Nutrients. 2018;10:1348. 30241426 10.3390/nu10101348 PMC6213862

[46] Shade MY Berger AM Dizona PJ Pozehl BJ Pullen CH. Sleep and health-related factors in overweight and obese rural women in a randomized controlled trial. J Behav Med. 2016;39:386–97. 26661065 10.1007/s10865-015-9701-y

[47] Sperry SD Scully ID Gramzow RH Jorgensen RS. Sleep duration and waist circumference in adults: a meta-analysis. Sleep. 2015;38:1269–76. 25581918 10.5665/sleep.4906 PMC4507732

[48] Polesel DN Hirotsu C Nozoe KT. Waist circumference and postmenopause stages as the main associated factors for sleep apnea in women: a cross-sectional population-based study. Menopause. 2015;22:835–44. 25668307 10.1097/GME.0000000000000406

[49] Rahman EU Chobufo MD Farah F. Prevalence and risk factors for the development of abdominal aortic calcification among the US population: NHANES study. Arch Med Sci Atheroscler Dis. 2021;6:e95–101. 34027218 10.5114/amsad.2021.105527 PMC8117070

[50] Fang H Xie F Li K Li M Wu Y. Association between weight-adjusted-waist index and risk of cardiovascular diseases in United States adults: a cross-sectional study. BMC Cardiovasc Disord. 2023;23:435. 37658325 10.1186/s12872-023-03452-z PMC10474739

[51] Zhang D Shi W Ding Z Park J Wu S Zhang J. Association between weight-adjusted-waist index and heart failure: results from National Health and Nutrition Examination Survey 1999-2018. Front Cardiovasc Med. 2022;9:1069146. 36588556 10.3389/fcvm.2022.1069146 PMC9794568

[52] Qin Z Chang K Yang Q Yu Q Liao R Su B. The association between weight-adjusted-waist index and increased urinary albumin excretion in adults: a population-based study. Front Nutr. 2022;9:941926. 36034904 10.3389/fnut.2022.941926 PMC9412203

[53] Li M Yu X Zhang W. The association between weight-adjusted-waist index and depression: results from NHANES 2005-2018. J Affect Disord. 2024;347:299–305. 38000467 10.1016/j.jad.2023.11.073

[54] Yin YH Zhou SY Lu DF. Higher waist circumference is associated with increased likelihood of female infertility: NHANES 2017-2020 results. Front Endocrinol (Lausanne). 2023;14:1216413. 37937052 10.3389/fendo.2023.1216413 PMC10627239

